# Circulating phospholipid species and internet addiction severity in Japanese adolescents: a pilot lipidomics study

**DOI:** 10.3389/fpsyt.2026.1751247

**Published:** 2026-02-16

**Authors:** Tomoki Kawahara, Yukako Tani, Nobutoshi Nawa, Shin Morioka, Hiroaki Kajiho, Junko Sasaki, Takehiko Sasaki, Takeo Fujiwara

**Affiliations:** 1 Department of Public Health, Institute of Science Tokyo, Tokyo, Japan; 2 Department of Clinical Information Applied Sciences, Institute of Science Tokyo, Tokyo, Japan; 3 Department of Biochemical Pathophysiology, Institute of Science Tokyo, Tokyo, Japan

**Keywords:** adolescents, internet addiction test, lipidomics, phosphatidylcholine, phosphatidylethanolamine, plasma

## Abstract

Internet addiction is increasing among adolescents, yet biological markers remain scarce. Lipid metabolism influences neurobehavior, but its association with internet addiction is unclear. Plasma concentrations of 150 phospholipid species from eight classes—phosphatidylinositol (PI), PI3P, PI4P, PI(3,5)P_2_, PI(4,5)P_2_, phosphatidylethanolamine (PE), phosphatidylcholine (PC), and PIP_3_—were quantified in 34 Japanese adolescents (mean ± SD age = 16.4 ± 0.5 y) using targeted LC-MS/MS with isotopic internal standards. Internet addiction severity was assessed by the 20-item Internet Addiction Test (IAT; range 0–100). Associations between lipid species and IAT scores were examined using linear regression adjusted for sex, with false-discovery-rate (FDR) correction for multiple testing. Several PE and PC species showed significant associations with IAT. In particular, PE 36:4 (β = 5.00, 95% CI 1.67–8.33, q = 0.042) and PE 36:2 (β = –1.33, 95% CI –2.29 to –0.38, q = 0.042) were among the strongest indicators. Additional suggestive associations were observed for PE 38:3, PE 40:5, and PC 38:3. Specific circulating PE and PC species are associated with internet addiction severity in Japanese adolescents. These preliminary findings highlight lipid dysregulation as a potential biomarker for problematic internet use and warrant confirmation in larger, longitudinal studies.

## Introduction

Internet addiction has become a global public-health concern. A recent meta-analysis that pooled data from >2 million individuals across 64 countries estimated that ~14% of the world’s population meets criteria for generalized Internet addiction, with higher figures for smartphone and social-media overuse (1). In Japan, longitudinal school-based data show an annual incidence of 22% for new-onset Internet addiction among adolescents aged 15–16 years (2). These incidences coincide with the developmental window in which reward sensitivity peaks and executive control is still maturing, underscoring the need to identify biological markers that might signal heightened vulnerability.

Phospholipids are integral to neuronal membrane architecture and signaling. Disturbances in both brain and peripheral lipid classes—particularly phosphatidylethanolamine (PE), phosphatidylcholine (PC), and phosphatidylinositol bisphosphate (PIP_2_)—have been documented across various psychiatric conditions. Reviews have highlighted altered phospholipid composition across mood, psychotic, and substance-use disorders (3). Experimentally, elevated platelet PIP_2_ levels have been observed in depressed bipolar patients (4), and brains of individuals with Alzheimer’s disease show marked reductions in PE plasmalogens (5). Circulating lysophosphatidic acid and lysophosphatidylcholine species have been linked to affective symptomatology, although findings remain equivocal (6). Importantly, untargeted lipidomics of young adults with Internet gaming disorder revealed specific PE and PC species that distinguished affected individuals from controls, suggesting a lipidome–behavior association that extends to digital-use phenotypes (7).

To date, no study has examined whether quantitative differences in canonical phospholipid fractions (PE, PC, and phosphoinositide derivatives) are associated with the severity of Internet addiction in a non-clinical youth cohort. The absence of data is notable, given that these phospholipids modulate synaptic plasticity and dopaminergic signaling pathways implicated in reward-seeking behaviors. Addressing this gap could yield potential biomarkers for early detection and mechanistic insights.

Using targeted quantitative lipidomics, we investigated the associations between plasma PE, PC, and phosphoinositide profiles and Internet Addiction Test (IAT) scores in Japanese adolescents aged 16–17 years. We hypothesized that specific phospholipid species would exhibit significant associations with IAT-defined Internet dependence after adjustment for socioeconomic, lifestyle, and psychosocial covariates.

## Methods

### Study design and population

This study recruited participants from the A-CHILD study and surveyed them when they were 16–17 years old (A-CHILD NEXT study). A consent form was mailed to the participants with the survey instructions and written consent was obtained. The subjects were minors, and consent was also obtained from their parent or caregivers. Cross-sectional data came from the A-CHILD NEXT follow-up (Adachi City, Tokyo). Of 34 participants with complete lipid and questionnaire data (2019 n = 14; 2021 n = 20), mean age was 16.4 y (SD 0.5); 59% were male. At the study visit, participants completed self-administered questionnaires and underwent fasting venous blood sampling. Participants who provided a blood sample received a 5000-yen honorarium. The Institute of Science Tokyo (formerly Tokyo Medical and Dental University) Ethics Committee approved the protocol (M2017-110); written assent and parental consent were obtained.

### Phospholipid profiling

Fasting venous blood samples were collected at the study site and processed to obtain plasma for phospholipid profiling. Fasted plasma phospholipids were quantified by targeted liquid chromatography–tandem mass spectrometry (LC–MS/MS) using a reverse-phase column for phosphatidylcholine (PC) and phosphatidylethanolamine (PE), and a chiral column for phosphoinositides.

For PC and PE measurements, 2 µL of plasma was mixed with 1.2 mL methanol containing 10 pmol each of C12:0/C13:0 PC and C12:0/C13:0 PE as internal standards, which possess odd-numbered fatty acyl chains absent in mammalian plasma and shorter chain lengths that yield distinct retention times from endogenous phospholipids. Subsequently, 600 µL ultrapure water, 600 µL 2 M HCl, and 100 µL 1 M NaCl were added. After thorough vortex-mixing, 2 mL chloroform was added, and the mixture was vortexed again for 2 min. The solution was centrifuged at 1200 × g for 4 min at room temperature, and the lower organic phase was collected and transferred to a fresh glass tube. The lipid extract was dried under a stream of nitrogen and reconstituted in 800 µL chloroform/methanol (1:1). For derivatization, 150 µL 0.6 M trimethylsilyl diazomethane was added to the fraction at room temperature. After 10 min, the reaction was quenched with 20 µL glacial acetic acid. The samples were mixed with 400 µL chloroform and 400 µL ultrapure water, vortexed for 1 min, and centrifuged at 1200 × g for 4 min. The lower phase was dried under nitrogen and redissolved in 400 µL methanol/ultrapure water (3:1). An aliquot (10 µL) was injected for analysis. Reverse-phase LC–MS/MS was performed using a QTRAP 6500 triple quadrupole mass spectrometer (AB SCIEX) coupled to a Nexera X2 HPLC system (Shimadzu) with a PAL HTC-xt autosampler (CTC Analytics). Separation of phospholipids employed an InertSustainBio C18 column (2.1 × 150 mm, 1.9 µm; GL Sciences) at 60 °C. The mobile phases consisted of (A) isopropanol/1 M ammonium acetate (200:1) and (B) methanol/acetonitrile/ultrapure water/1 M ammonium acetate (90:90:20:1). The flow rate was 100 µL/min with the following gradient: 0–2 min, 10% A; 2–4 min, 10–40% A; 4–11 min, 40–55% A; 11–17 min, 55–82% A; 17–21 min, 82% A; and 21–29 min, 10% A. Multiple reaction monitoring (MRM) transitions (precursor/product ion pairs) are listed in **Supplementary Table 2**. Data were acquired using Analyst 1.6.3 (AB SCIEX) and processed in MultiQuant (AB SCIEX) for manual peak integration. No background subtraction was applied. Gaussian smoothing width was set to 1.0 point. Quantification was based on the ratio of sample peak area to internal standard peak area.

For phosphoinositide [PI, PI3P, PI4P, PI (3,5)P_2_, PI (4,5)P_2_, and PIP_3_] analyses, lipid extraction was performed as described previously (8). 100 µL plasma was mixed with 1.5 mL methanol. To this suspension, 50 µL of methanol/chloroform (9:1) containing 1 nmol C8:0/C8:0 PI (4,5)P_2_ (used as an adsorption inhibitor) and 10 pmol each of synthetic C17:0/C20:4 phosphoinositides (internal standards) were added. Subsequently, 750 µL ultrapure water, 750 µL 2 M HCl, and 200 µL 1 M NaCl were added. After vortex-mixing, 3 mL chloroform was added, followed by an additional 2 min vortex. The mixture was centrifuged at 1200 × g for 4 min at room temperature. The lower organic phase containing crude lipids was transferred to a clean glass tube. Phosphoinositides were preconcentrated using a DEAE Sepharose Fast Flow anion-exchange resin (10% slurry). The resin was sequentially washed twice with ultrapure water, once with 1 M HCl, twice with water, once with 1 M NaOH, and twice again with water, then resuspended in methanol to form a 50% slurry. A 0.5 mL bed volume was packed into a Pasteur pipette plugged with glass wool. The crude lipid extract (2.9 mL) mixed with 1.5 mL methanol was applied to the column. The column was washed with 3 mL chloroform/methanol (1:1) and 2 mL chloroform/methanol/28% aqueous ammonia/glacial acetic acid (200:100:3:0.9). Elution was performed with 1.5 mL chloroform/methanol/12 M HCl/ultrapure water (12:12:1:1). The eluted fraction was mixed with 850 µL 120 mM NaCl and centrifuged at 1200 × g for 4 min at room temperature. The lower phase containing purified phosphoinositides was collected. For derivatization, 150 µL 0.6 M trimethylsilyl diazomethane was added to the purified fraction at room temperature. After 10 min, the reaction was quenched with 20 µL glacial acetic acid. The samples were mixed with 700 µL methanol/ultrapure water/chloroform (48:47:3), vortexed for 1 min, and centrifuged at 1200 × g for 4 min. The lower phase was dried under nitrogen and redissolved in 100 µL acetonitrile. LC–MS/MS analysis was performed as described above for PC and PE analysis. A 10 µL aliquot was injected, and separation was achieved on a CHIRALPAK IC-3 column (2.1 × 250 mm, 3 µm; DAICEL) at 22 °C. Chromatography was carried out at a flow rate of 100 µL/min with the following gradient: 40% mobile phase A (methanol/5 mM ammonium acetate) and 60% mobile phase B (acetonitrile/5 mM ammonium acetate) for 1 min, linearly increasing to 85% A over 2 min, and held at 85% A for 11 min. MRM transitions are listed in **Supplementary Table 3**. Acquired data were analyzed using Analyst 1.6.3 (AB SCIEX) and MultiQuant (AB SCIEX), as described above for PC and PE analysis. Features failing laboratory quality control were excluded. Transitions not meeting laboratory quality control criteria, such as undetectable peaks at the noise level, were excluded from quantification, and their values were assigned as zero. For each molecular species, abundance was expressed as the ratio of analyte peak area to its isotopic internal standard (arbitrary units); no external calibration was performed. Reported values therefore represent internal−standard–normalized intensities.

### Internet-addiction test

The 20-item IAT (range 0–100; validated Japanese version) was administered using the validated Japanese translation (JIAT) (9, 10). The primary outcome was the continuous IAT score. For descriptive categorization, IAT scores ≥40 were treated as at least moderate dependence, consistent with the conventional thresholds adopted in Japan and prior work using the IAT (11).

### Statistical analysis

Analyses used Python 3.11.10 (pandas 2.2.2; statsmodels 0.14). For each lipid species within each class, we fit a sex-adjusted ordinary least-squares model with IAT as the outcome (complete-case per lipid). We report the regression coefficient ( β per unit of lipid abundance), 95% CI, and p-value. Multiple testing was controlled within lipid class using Benjamini–Hochberg false-discovery rate (FDR); q<0.05 indicated statistical evidence and 0.05≤q<0.30 was treated as exploratory (12). We controlled multiplicity within lipid class using the Benjamini–Hochberg procedure. The number of tests (m) per class was: PI (m = 16), PI3P (12), PI4P (16), PI(3,5)P_2_ (17), PI(4,5)P_2_ (16), PIP_3_ (5), PC (15), and PE (15). β reflects the mean difference in IAT score per one−unit increase in the IS−normalized ratio (a.u.). Figures comprise a volcano plot of β versus −log10(q) and a two-panel display (scatter with fitted line; IAT by lipid quartiles) for the lipid with the smallest q. Two-sided α=0.05.

### Ethics statement

The A−CHILD NEXT follow−up was approved by the Institute of Science Tokyo Research Ethics Committee (M2017−110). Parents or legal guardians provided written informed consent; adolescents provided written assent before any study procedures. No incentives were contingent on survey responses, and participation could be discontinued at any time without penalty.

## Results

### Participant characteristics

Among 34 adolescents (59% male; mean ± SD age 16.4 ± 0.5 years), the mean Internet-Addiction Test (IAT) score was 47.9 ± 13.4 (range 24–71). Two participants scored ≥70, which is classified as “severe” on the Internet Addiction Test and indicates that Internet use may cause major problems in daily life (13). Using the descriptive threshold of IAT ≥40, 28/34 (82%) met criteria for at least moderate dependence. IAT did not differ by sex (p=0.91). Parental divorce or bereavement was uncommon (18%), and most households reported annual income ≥ 600,000 JPY (**Supplementary Table 1**).

### Lipid data and modeling

After quality control, 138 of 150 phospholipid species were analyzed. For each species, a sex-adjusted ordinary least-squares model estimated the association with continuous IAT; multiplicity was controlled within class using Benjamini–Hochberg. Lipid species with q<0.30 are summarized in **Table 1**. Multivariate analysis revealed that while principal component analysis (PCA) showed overlapping distributions, partial least squares discriminant analysis (PLS-DA) indicated a trend of separation by IAT severity (**Supplementary Figure S1**).

**Table 1:** Lipid species associated with Internet Addiction Tendency (IAT).

Class	Lipid	β	CI_low	CI_high	p	q
PC	PC 38:3	10.32	3.25	17.39	<0.001	0.084
PC	PC 40:4	66.45	14.18	118.73	0.014	0.108
PE	PE 36:2	-1.33	-2.29	-0.38	0.008	0.042
PE	PE 36:4	5.00	1.67	8.33	0.005	0.042
PE	PE 38:3	9.77	2.39	17.16	0.011	0.042
PE	PE 40:5	13.84	3.47	24.21	0.011	0.042
PE	PE 40:4	18.98	0.60	37.37	0.043	0.130
PE	PE 32:1	40.79	-6.18	87.75	0.086	0.216
PE	PE 36:3	-3.39	-7.52	0.74	0.104	0.223

Linear regression models were fitted for IAT on each lipid species, adjusted for sex. The table shows the lipid class, outcome (IAT), lipid identifier, regression coefficient (β), 95% confidence interval (CI), p-value, and Benjamini–Hochberg adjusted q-value within lipid class. Positive β indicates higher IAT scores with increasing lipid concentration. q: Benjamini–Hochberg FDR within lipid class (m as specified in Methods). Lipids with q< 0.05 are considered statistically significant, while those with q< 0.30 are highlighted as suggestive associations.

### Findings concentrated in PE and PC

Four phosphatidylethanolamines met FDR q<0.05 (**Table 1**): PE 36:2 showed an inverse association with IAT (β = −1.33; 95% CI, −2.29 to −0.38; p=0.008; q=0.042), whereas PE 36:4 (β = 5.00; 1.67 to 8.33; p=0.005; q=0.042), PE 38:3 (β = 9.77; 2.39 to 17.16; p=0.011; q=0.042), and PE 40:5 (β = 13.84; 3.47 to 24.21; p=0.011; q=0.042) were positively associated. Within phosphatidylcholines, PC 38:3 was suggestive (**Table 1**; β = 10.32; 3.25 to 17.39; p<0.001; q=0.084). PC 40:4 had a large point estimate but wide uncertainty (β = 66.45; 14.18 to 118.73; p=0.014; q=0.108), consistent with sparse information. Additional PE species (e.g., PE 40:4, PE 32:1, PE 36:3) were exploratory (0.05≤q<0.30). Signals outside PE/PC did not meet the pre-specified q<0.05 threshold.

**Figure 1:**
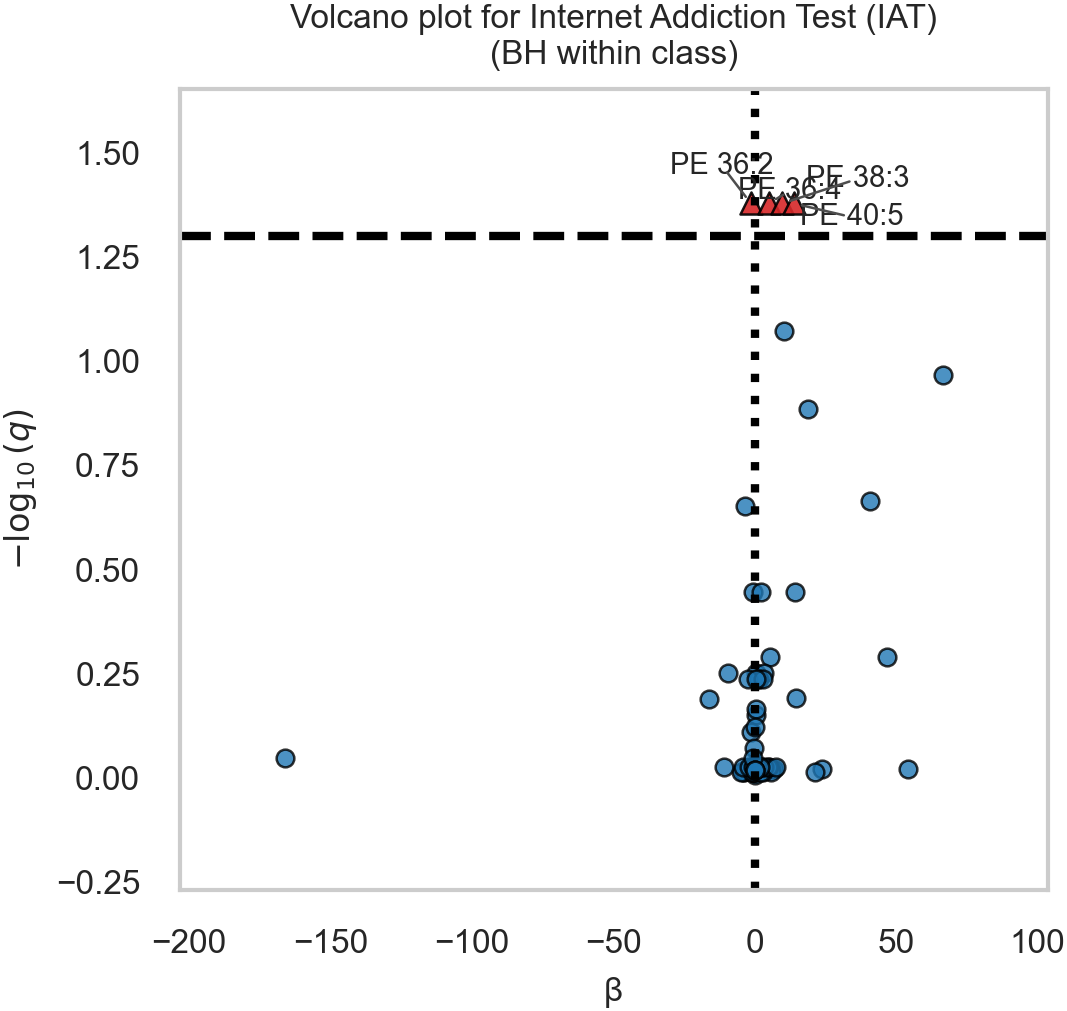
Plasma phospholipid concentration distributions stratified by internet-addiction tendency (IAT < 40 vs ≥ 40).Distribution of plasma phospholipid concentrations by Internet-addiction status (IAT < 40 vs ≥ 40). Boxes show the median and interquartile range (IQR); whiskers extend to 1.5 × IQR; dots represent outliers. Blue boxes correspond to non-addicted participants and red boxes to addicted participants. Statistical contrasts are unadjusted.

**Figure 2:**
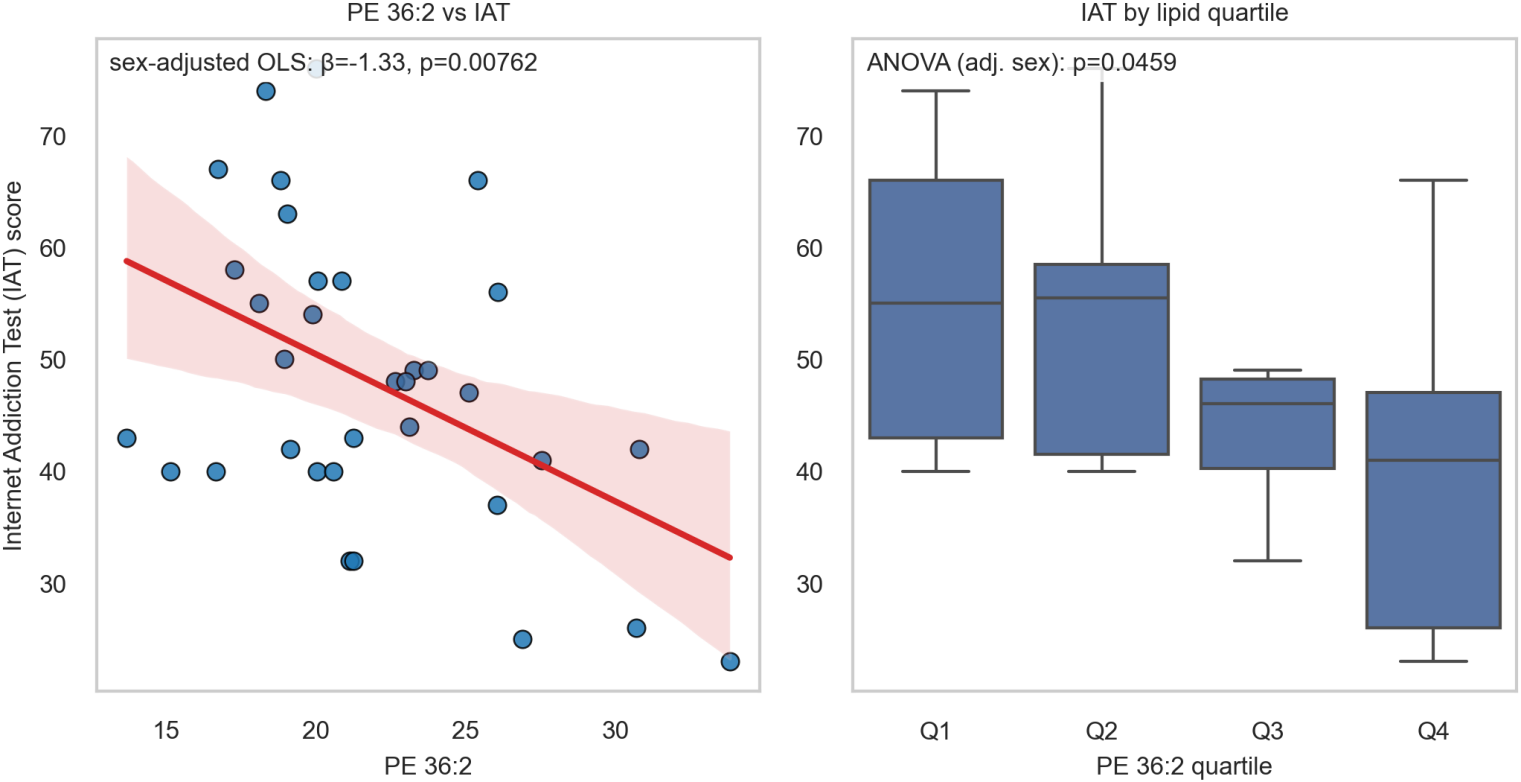
Volcano plot of lipid–internet addiction tendency (IAT) associations. This figure shows the β coefficients from linear regression models of lipids predicting Internet Addiction Tendency (IAT). The x-axis indicates standardized β, and the y-axis shows the negative logarithm of the Benjamini–Hochberg adjusted q-value (–log10 q). Each dot represents one lipid species, colored red when q ≤ 0.05 within its lipid class and grey otherwise. Dashed horizontal line corresponds to the significance threshold (q = 0.05). Labels are displayed for significant lipids.

**Figure 3:**
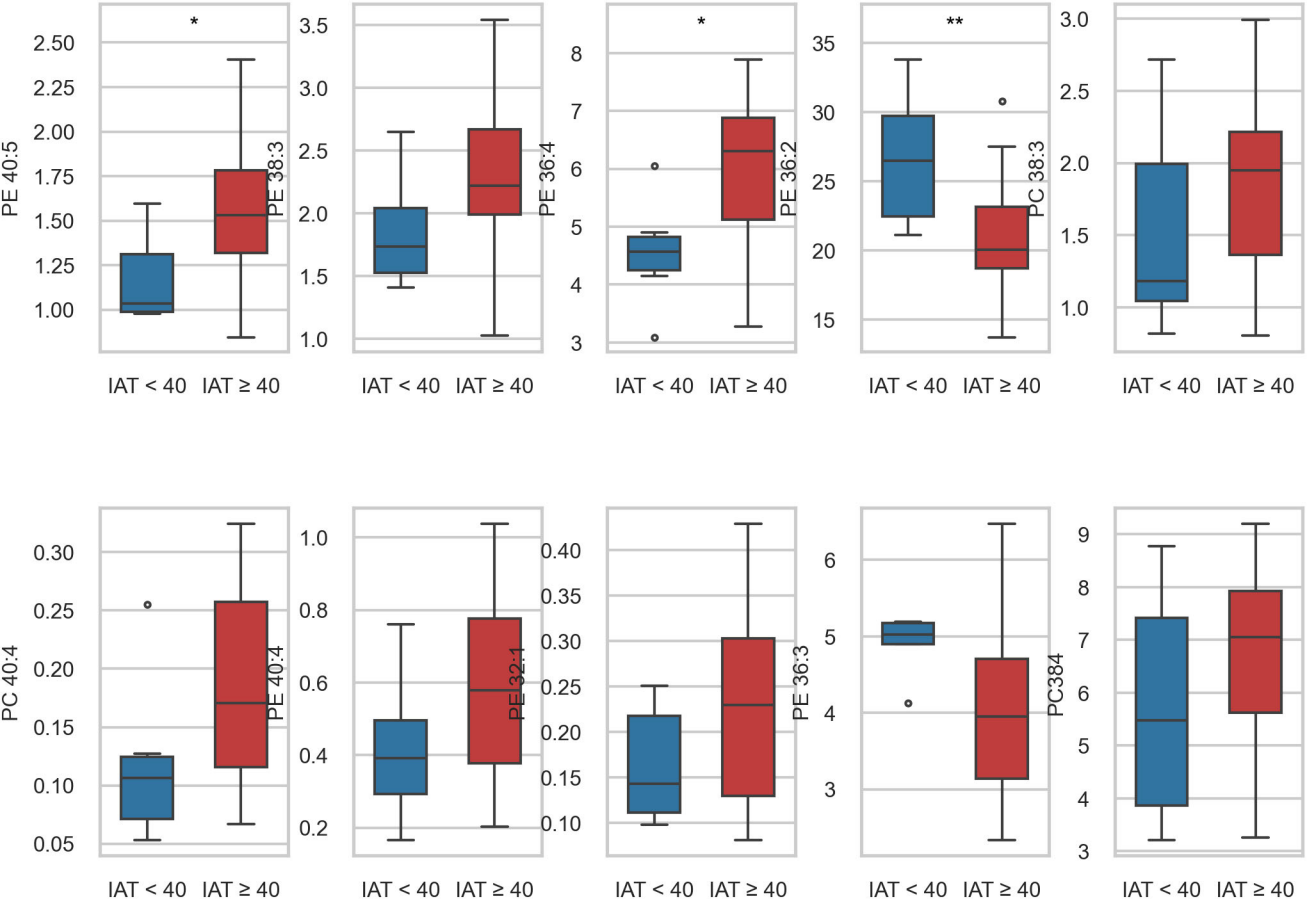
Key lipid associated with internet addiction tendency (IAT). The lipid species with the smallest q-value is illustrated. Left panel: scatterplot with fitted ordinary least squares regression line showing the association between the lipid concentration and IAT score. Right panel: box-and-whisker plot of IAT across quartiles of the lipid. The x-axis indicates lipid quantiles (Q1–Q4), and the y-axis indicates IAT score. Together, these plots highlight the strongest observed lipid–IAT association. ** p ≤ 0.01; * p ≤ 0.05.

The volcano plot (**Figure 1**) summarizes estimates and adjusted evidence across species (red points: q<0.05). **Figure 2** illustrates the lipid with the minimum q (ties resolved programmatically), showing the scatter with fitted line and IAT across lipid quartiles, and **Figure 3** further depicts this strongest lipid–IAT association using the same two-panel display (scatterplot and IAT by lipid quartiles).

## Discussion

In this cross-sectional pilot of Japanese adolescents (n=34), sex-adjusted per-lipid analyses identified several PE species that were associated with higher IAT scores—PE 36:4, PE 38:3, and PE 40:5—and one PE species inversely associated (PE 36:2). A PC species (PC 38:3) showed suggestive evidence. Signals centered on PE, with smaller contributions from PC, while other classes did not pass the pre-specified false-discovery threshold.

Prior lipidomics and membrane-lipid studies in neuropsychiatry have described altered phospholipid profiles across mood and cognitive conditions, and a study in internet gaming disorder reported disturbed lipidomic patterns in young men (3, 7). Our class-specific pattern—concentrated in select PE species with one PC species suggestive—extends this literature to a community adolescent sample assessed with the IAT and is consistent with variation in membrane phospholipid composition across behavioral phenotypes. Differences in age, sex composition, biospecimens, platforms, and adjustment sets may account for discrepancies across studies.

Notably, in addition to four PE species (36:4, 38:3, and 40:5), one PC species (38:3) also emerged as a promising correlate of Internet addiction severity. This overlap is biologically plausible, given the metabolic connectivity between PE and PC. In mammalian cells, PE can be sequentially methylated to PC via the phosphatidylethanolamine N-methyltransferase (PEMT) pathway, which is particularly active in neuronal tissues and in the liver. Conversely, both PC and PE serve as substrates for phosphatidylserine synthases (PSS1 and PSS2), which catalyze base-exchange reactions with serine to produce phosphatidylserine (PS); PS can subsequently be decarboxylated back to PE within mitochondria. These interlinked reactions establish a bidirectional metabolic flux between PE and PC, suggesting that perturbations in one phospholipid pool may propagate to the other. Thus, the parallel associations of PE and PC molecular species—especially the shared 38:3 species—observed here may reflect coordinated remodeling or compensatory homeostatic regulation within the membrane phospholipid network, rather than isolated lipid changes.

In addition to differences among phospholipid classes defined by their hydrophilic headgroups—that is, PE and PC—the present findings underscore the importance of acyl-chain diversity within each class. A strength of this study lies in its acyl-species-level resolution, which enables the identification of functionally distinct lipid species that share the same headgroup but differ in acyl composition. In neuronal cells, phospholipids play pivotal roles in maintaining membrane fluidity, curvature, and vesicle fusion, processes through which receptor functions and intracellular signaling are dynamically regulated (13, 14). These biophysical properties are critically shaped by the acyl-chain composition of individual phospholipid species. Within each phospholipid class, molecular species differ in the length and saturation of their fatty acyl chains, leading to distinct physical behaviors in the membrane environment. Glycerophospholipids undergo continuous acyl-chain remodeling through the Lands cycle—a conserved two-step process involving phospholipase A_2_ (PLA_2_)–mediated deacylation to generate lysophospholipids, followed by reacylation catalyzed by specific lysophospholipid acyltransferases (LPLATs), including ysophosphatidylcholine acyltransferase (LPCAT) and Lysophosphatidylethanolamine acyltransferase (LPEAT). This remodeling fine-tunes the acyl composition of membrane phospholipid species, thereby modulating membrane curvature, vesicle fusion efficiency, and receptor-mediated signaling. In addition, glycerophospholipids serve as reservoirs for signaling precursors and sources of bioactive lipid mediators: for example, phospholipase C and D (PLC/PLD) reactions generate diacylglycerol (DAG) and phosphatidic acid (PA), while PLA_2_ activity releases arachidonic acid for eicosanoid synthesis and produces diverse lysophospholipid signaling molecules (15–19). Both the resulting changes in membrane biophysics and in lipid-derived signaling cascades represent processes that underlie synaptic plasticity and may be relevant to the neurobiological alterations associated with Internet addiction.

Against this mechanistic backdrop, the observed pattern—positive associations for multiple PE species, represented by PE 38:3, and for PC 38:3, contrasted with an inverse association for PE 36:2—may reflect heterogeneous remodeling states rather than a single pathway (14–17). Such acyl-specific variability likely arises from differences in the enzymatic selectivity or subcellular localization of acyltransferases operating within the Lands cycle. Clinically, while this phospholipid panel is not diagnostic, replication of these associations could guide hypothesis generation, research-oriented risk stratification, and targeted mechanistic experiments.

The sample was small, thus the precision and generalizability are limited. The design was cross-sectional, precluding temporality, that is, IAT may induce some eating behaviors (cite), which reflect as phospholipid profile; alternatively, phospholipid profile may form IAT. IAT was self-reported, and only sex was used for covariate adjustment; residual confounding (e.g., genetic predisposition) is possible. Although multiplicity was controlled within class using Benjamini–Hochberg, false positives remain possible and several estimates were imprecise (12). Batch and technical effects could not be fully evaluated. Findings should be considered exploratory.

In conclusion, specific PE species—and to a lesser extent PC 38:3—showed the strongest associations with IAT in this pilot. Replication in larger, preregistered, multi-center cohorts with longitudinal designs, richer adjustment, and standardized lipidomics is needed. Future experimental investigations should further elucidate how PE remodeling influences neural signaling and the potential bidirectional interactions between circulating phospholipids and the brain.

## Data Availability

Data are available upon reasonable request, subject to approval by the authors’ Institutional Ethics Committee.
