# Supplementary material for: Circulating phospholipid species and internet addiction severity in Japanese adolescents: a pilot lipidomics study
Source: Front Psychiatry. 2026 Feb 16;17:1751247. doi: 10.3389/fpsyt.2026.1751247 (PMC12950673; doi:10.3389/fpsyt.2026.1751247)
Supplement: Supplementary file 1 [file DataSheet1.docx]

**Supplementary Table 1.** Participant characteristics and covariates in the A-CHILD NEXT cohort, overall and stratified by internet addiction tendency (n = 34).

|  | non-addicted | addicted | p-value |
| --- | --- | --- | --- |
|  | N=6 | N=28 |  |
| Age | 16.7 (0.5) | 16.4 (0.5) | 0.23 |
| Sex |  |  | 0.63 |
| Male | 3 (50.0%) | 17 (60.7%) |  |
| Female | 3 (50.0%) | 11 (39.3%) |  |
| Parents' marital status |  |  | 0.89 |
| Married/de facto marriage | 5 (83.3%) | 23 (82.1%) |  |
| Divorced | 1 (16.7%) | 4 (14.3%) |  |
| Bereavement | 0 (0.0%) | 1 (3.6%) |  |
| Annual household income |  |  | 0.25 |
| 200-399 | 0 (0%) | 3 (11%) |  |
| 400-599 | 3 (50%) | 7 (25%) |  |
| 600-749 | 3 (50%) | 5 (18%) |  |
| 750-999 | 0 (0%) | 3 (11%) |  |
| >1000 | 0 (0%) | 6 (21%) |  |
| Missing/No answer | 0 (0%) | 4 (14%) |  |
| Have a dream for the future |  |  | 0.95 |
| Yes | 4 (66.7%) | 19 (67.9%) |  |
| No | 2 (33.3%) | 9 (32.1%) |  |
| Self-esteem scale | 33.5 (9.9) | 31.0 (8.8) | 0.54 |
| Subjective happiness | 7.3 (3.4) | 6.9 (1.6) | 0.60 |
| Flexibility Scale of Cognition in College Students' Version | 3.5 (0.9) | 3.0 (1.1) | 0.32 |
| Coping scale | 23.0 (7.9) | 23.5 (4.2) | 0.81 |
| Experience of being bullied |  |  | 0.31 |
| Yes | 0 (0.0%) | 4 (14.3%) |  |
| No | 6 (100.0%) | 23 (82.1%) |  |
| Missing | 0 (0.0%) | 1 (3.6%) |  |
| Sleeping hours on weekdays |  |  | 0.23 |
| 8-9h/day | 1 (16.7%) | 3 (10.7%) |  |
| 7-8h/day | 3 (50.0%) | 5 (17.9%) |  |
| 6-7h/day | 2 (33.3%) | 11 (39.3%) |  |
| <6h/day | 0 (0.0%) | 9 (32.1%) |  |
| Frequency of breakfast intake |  |  | 0.60 |
| Take everyday | 5 (%) | 20 (%) |  |
| Not take everyday | 0 (%) | 3 (%) |  |
| Most do not take | 0 (%) | 3 (%) |  |
| Do not always take | 1 (%) | 2 (%) |  |
| Number of books read in a month |  |  | 0.48 |
| 0 | 4 (66.7%) | 13 (46.4%) |  |
| 1 | 0 (0.0%) | 7 (25.0%) |  |
| 2-3 | 1 (16.7%) | 6 (21.4%) |  |
| 4-7 | 1 (16.7%) | 2 (7.1%) |  |
| Whether like to read or not |  |  | 0.28 |
| very likeable | 1 (16.7%) | 3 (10.7%) |  |
| like | 2 (33.3%) | 15 (53.6%) |  |
| do not like much | 3 (50.0%) | 5 (17.9%) |  |
| do not like | 0 (0.0%) | 5 (17.9%) |  |
| Subjective health status |  |  | 0.54 |
| Good | 2 (33.3%) | 5 (17.9%) |  |
| So-so | 0 (0.0%) | 7 (25.0%) |  |
| Normal | 2 (33.3%) | 9 (32.1%) |  |
| Not very good | 2 (33.3%) | 5 (17.9%) |  |
| Not good | 0 (0.0%) | 2 (7.1%) |  |

Continuous variables are presented as mean ± SD; categorical variables are shown as n (%). Age is reported in years; Rosenberg self-esteem score ranges from 10 to 40 (higher = higher self-esteem). Household income denotes annual pretax income in thousands of Japanese yen. Week-day sleep duration, breakfast frequency, and tooth-brushing frequency were self-reported in the categories shown. Bullying experience refers to any episode of being bullied since age 12. The Internet-Addiction Test (IAT) ranges from 0 to 100; scores ≥40 indicate at least moderate dependence. P-values compare IAT <40 vs ≥40 by Student’s t-test (continuous) or Fisher’s exact test (categorical).

**Supplementary Table 2.** Phosphatidylcholine (PC) and Phosphatidylethanolamine (PE) multiple-reaction-monitoring (MRM) transitions.

| Molecular species | Q1 (m/z) | Q3 (m/z) | Collision energy (eV) |
| --- | --- | --- | --- |
| 25:0 PE | 608.57 | 453.5 | 32 |
| 32:0 PE | 706.57 | 551.5 | 34 |
| 32:1 PE | 704.57 | 549.5 | 34 |
| 34:0 PE | 734.57 | 579.5 | 34 |
| 34:1 PE | 732.57 | 577.5 | 34 |
| 34:2 PE | 730.57 | 575.5 | 34 |
| 36:0 PE | 762.57 | 607.5 | 35 |
| 36:1 PE | 760.57 | 605.5 | 35 |
| 36:2 PE | 758.57 | 603.5 | 35 |
| 36:3 PE | 756.57 | 601.5 | 35 |
| 36:4 PE | 754.57 | 599.5 | 35 |
| 38:3 PE | 784.57 | 629.5 | 35 |
| 38:4 PE | 782.57 | 627.5 | 36 |
| 38:5 PE | 780.57 | 625.5 | 36 |
| 38:6 PE | 778.57 | 623.5 | 35 |
| 40:4 PE | 810.57 | 655.5 | 35 |
| 40:5 PE | 808.57 | 653.5 | 35 |
| 40:6 PE | 806.57 | 651.5 | 35 |
| 25:0 PC | 650.62 | 198.1 | 36 |
| 32:0 PC | 748.62 | 198.1 | 38 |
| 32:1 PC | 746.62 | 198.1 | 38 |
| 34:0 PC | 776.62 | 198.1 | 38 |
| 34:1 PC | 774.62 | 198.1 | 38 |
| 34:2 PC | 772.62 | 198.1 | 38 |
| 36:0 PC | 804.62 | 198.1 | 39 |
| 36:1 PC | 802.62 | 198.1 | 39 |
| 36:2 PC | 800.62 | 198.1 | 39 |
| 36:3 PC | 798.62 | 198.1 | 39 |
| 36:4 PC | 796.62 | 198.1 | 39 |
| 38:3 PC | 826.62 | 198.1 | 39 |
| 38:4 PC | 824.62 | 198.1 | 39 |
| 38:5 PC | 822.62 | 198.1 | 39 |
| 38:6 PC | 820.62 | 198.1 | 40 |
| 40:4 PC | 852.62 | 198.1 | 40 |
| 40:5 PC | 850.62 | 198.1 | 40 |
| 40:6 PC | 848.62 | 198.1 | 40 |

List of Q1 → Q3 transitions, collision energies, and precursor/product ion pairs used for targeted quantification of PC and PE species by LC–MS/MS (QTRAP 6500, AB SCIEX). All transitions were acquired in positive-ion mode following derivatization with trimethylsilyl diazomethane. CE = collision energy (eV). Analyte intensities were normalized to the isotopic internal standards C12:0/C13:0 PE or C12:0/C13:0 PC.

**Supplementary Table 3.** Phosphoinositide multiple-reaction-monitoring (MRM) transitions.

| Molecular species | Q1 (m/z) | Q3 (m/z) | Collision energy (eV) |
| --- | --- | --- | --- |
| 31:1 PI | 826.7 | 535.5 | 32 |
| 32:0 PI | 842.7 | 551.5 | 32 |
| 32:1 PI | 840.7 | 549.5 | 32 |
| 34:0 PI | 870.7 | 579.5 | 32 |
| 34:1 PI | 868.7 | 577.5 | 32 |
| 34:2 PI | 866.7 | 575.5 | 32 |
| 36:0 PI | 898.7 | 607.5 | 33 |
| 36:1 PI | 896.7 | 605.5 | 33 |
| 36:2 PI | 894.7 | 603.5 | 33 |
| 36:3 PI | 892.7 | 601.5 | 33 |
| 36:4 PI | 890.7 | 599.5 | 33 |
| 38:3 PI | 920.7 | 629.5 | 34 |
| 38:4 PI | 918.7 | 627.5 | 34 |
| 38:5 PI | 916.7 | 625.5 | 34 |
| 38:6 PI | 914.7 | 623.5 | 34 |
| 40:4 PI | 946.7 | 655.5 | 35 |
| 40:5 PI | 944.7 | 653.5 | 35 |
| 40:6 PI | 942.7 | 651.5 | 35 |
| 37:4 PIP1 | 1012.7 | 613.5 | 37 |
| 32:0 PIP1 | 950.7 | 551.5 | 35 |
| 32:1 PIP1 | 948.7 | 549.5 | 35 |
| 34:0 PIP1 | 978.7 | 579.5 | 35 |
| 34:1 PIP1 | 976.7 | 577.5 | 35 |
| 34:2 PIP1 | 974.7 | 575.5 | 35 |
| 36:0 PIP1 | 1006.7 | 607.5 | 36 |
| 36:1 PIP1 | 1004.7 | 605.5 | 36 |
| 36:2 PIP1 | 1002.7 | 603.5 | 36 |
| 36:3 PIP1 | 1000.7 | 601.5 | 36 |
| 36:4 PIP1 | 998.7 | 599.5 | 36 |
| 38:3 PIP1 | 1028.7 | 629.5 | 37 |
| 38:4 PIP1 | 1026.7 | 627.5 | 37 |
| 38:5 PIP1 | 1024.7 | 625.5 | 37 |
| 38:6 PIP1 | 1022.7 | 623.5 | 37 |
| 40:4 PIP1 | 1054.7 | 655.5 | 38 |
| 40:5 PIP1 | 1052.7 | 653.5 | 38 |
| 40:6 PIP1 | 1050.7 | 651.5 | 38 |
| 37:4 PIP2 | 1120.7 | 613.5 | 38 |
| 32:0 PIP2 | 1058.7 | 551.5 | 36 |
| 32:1 PIP2 | 1056.7 | 549.5 | 36 |
| 34:0 PIP2 | 1086.7 | 579.5 | 36 |
| 34:1 PIP2 | 1084.7 | 577.5 | 36 |
| 34:2 PIP2 | 1082.7 | 575.5 | 36 |
| 36:0 PIP2 | 1114.7 | 607.5 | 37 |
| 36:1 PIP2 | 1112.7 | 605.5 | 37 |
| 36:2 PIP2 | 1110.7 | 603.5 | 37 |
| 36:3 PIP2 | 1108.7 | 601.5 | 37 |
| 36:4 PIP2 | 1106.7 | 599.5 | 37 |
| 38:3 PIP2 | 1136.7 | 629.5 | 38 |
| 38:4 PIP2 | 1134.7 | 627.5 | 38 |
| 38:5 PIP2 | 1132.7 | 625.5 | 38 |
| 38:6 PIP2 | 1130.7 | 623.5 | 38 |
| 40:4 PIP2 | 1162.7 | 655.5 | 39 |
| 40:5 PIP2 | 1160.7 | 653.5 | 39 |
| 40:6 PIP2 | 1158.7 | 651.5 | 39 |
| 37:4 PIP3 | 1228.7 | 613.5 | 40 |
| 32:0 PIP3 | 1166.7 | 551.5 | 38 |
| 32:1 PIP3 | 1164.7 | 549.5 | 38 |
| 34:0 PIP3 | 1194.7 | 579.5 | 38 |
| 34:1 PIP3 | 1192.7 | 577.5 | 38 |
| 34:2 PIP3 | 1190.7 | 575.5 | 38 |
| 36:0 PIP3 | 1222.7 | 607.5 | 39 |
| 36:1 PIP3 | 1220.7 | 605.5 | 39 |
| 36:2 PIP3 | 1218.7 | 603.5 | 39 |
| 36:3 PIP3 | 1216.7 | 601.5 | 39 |
| 36:4 PIP3 | 1214.7 | 599.5 | 39 |
| 38:3 PIP3 | 1244.7 | 629.5 | 40 |
| 38:4 PIP3 | 1242.7 | 627.5 | 40 |
| 38:5 PIP3 | 1240.7 | 625.5 | 40 |
| 38:6 PIP3 | 1238.7 | 623.5 | 40 |
| 40:4 PIP3 | 1270.7 | 655.5 | 41 |
| 40:5 PIP3 | 1268.7 | 653.5 | 41 |
| 40:6 PIP3 | 1266.7 | 651.5 | 41 |

Q1 → Q3 transitions, collision energies, and fragment ions used for quantification of phosphoinositide classes—PI, PIP_1_ (monophosphate), PIP_2_ (bisphosphate), and PIP_3_ (trisphosphate)—in plasma by LC–MS/MS (QTRAP 6500, AB SCIEX) operated in negative-ion mode. CE = collision energy (eV). Synthetic C17:0/C20:4 phosphoinositide analogues were used as internal standards for each subclass.

**Supplementary Figure S1.** Multivariate analysis of plasma lipidomics data.


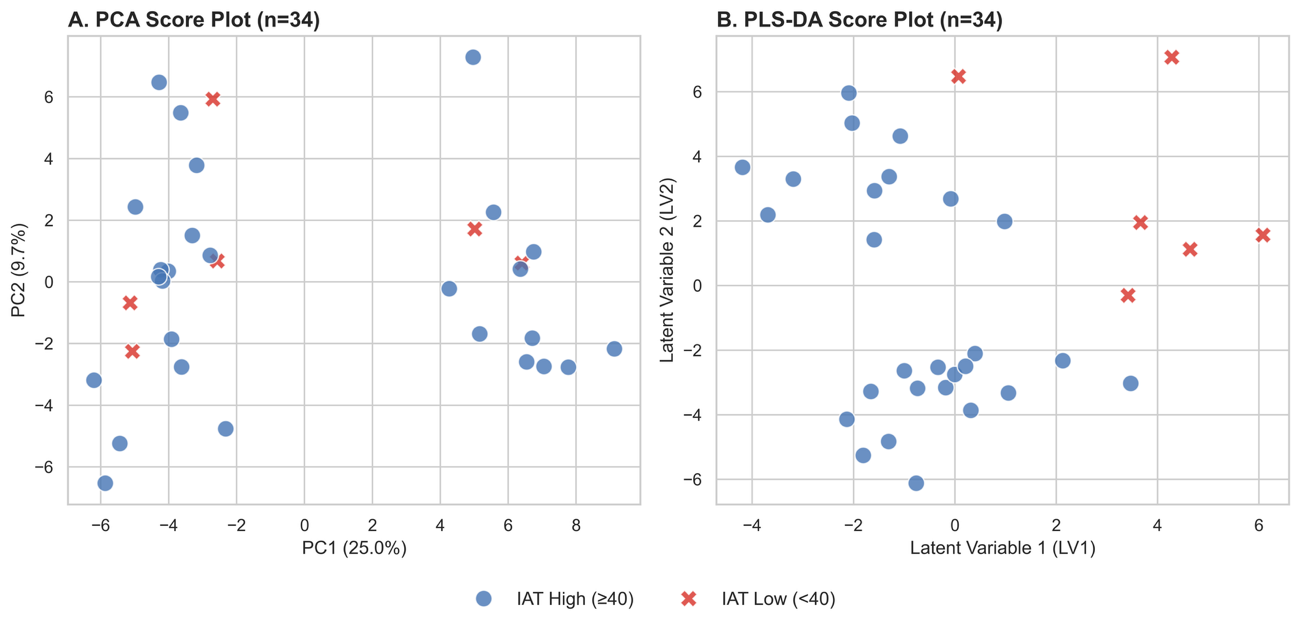


(A) Principal Component Analysis (PCA) score plot and (B) Partial Least Squares Discriminant Analysis (PLS-DA) score plot of the 115 phospholipid species quantified in Japanese adolescents (N=34). Participants are color-coded by Internet Addiction Test (IAT) severity (Blue: IAT < 40, Low/Normal; Red: IAT $\geq$ 40, Moderate/Severe). While the unsupervised PCA shows overlapping distributions, suggesting no global alteration in the lipidome, the supervised PLS-DA indicates a trend of separation, consistent with the presence of specific lipid species associated with IAT severity.
